# *Wolbachia* Infection in a Natural Parasitoid Wasp Population

**DOI:** 10.1371/journal.pone.0134843

**Published:** 2015-08-05

**Authors:** Anne Duplouy, Christelle Couchoux, Ilkka Hanski, Saskya van Nouhuys

**Affiliations:** 1 University of Helsinki, Metapopulation Research Centre, Department of Biosciences, P.O. Box 65, FI-00014, Helsinki, Finland; 2 University of Sussex, School of Life Sciences, Brighton BN19QG, United Kingdom; 3 Cornell University, Department of Ecology and Evolutionary Biology, Ithaca, New York, 14853, United States of America; International Atomic Energy Agency, AUSTRIA

## Abstract

The maternally transmitted bacterium *Wolbachia pipientis* is well known for spreading and persisting in insect populations through manipulation of the fitness of its host. Here, we identify three new *Wolbachia pipientis* strains, *w*Hho, *w*Hho2 and *w*Hho3, infecting *Hyposoter horticola*, a specialist wasp parasitoid of the Glanville fritillary butterfly. The *w*Hho strain (ST435) infects about 50% of the individuals in the Åland islands in Finland, with a different infection rate in the two mitochondrial (COI) haplotypes of the wasp. The vertical transmission rate of *Wolbachia* is imperfect, and lower in the haplotype with lower infection rate, suggesting a fitness trade-off. We found no association of the *w*Hho infection with fecundity, longevity or dispersal ability of the parasitoid host. However, preliminary results convey spatial associations between *Wolbachia* infection, host mitochondrial haplotype and parasitism of *H*. *horticola* by its hyperparasitoid, *Mesochorus* cf. *stigmaticus*. We discuss the possibility that *Wolbachia* infection protects *H*. *horticola* against hyperparasitism.

## Introduction

*Wolbachia pipientis* is estimated to infect up to 60% of all insect species [1–4]. The maternally inherited bacterial endosymbiont often manipulates host reproduction to increase the frequency of infected host individuals in the population [5]. Moreover, *Wolbachia* can manipulate other aspects of host biology via interactions ranging from parasitism to mutualism. In parasitic interactions, *Wolbachia* infection decreases host fecundity [6–10], longevity [11], and other comparable physiological traits [12, 13], while in mutualistic interactions *Wolbachia* increases fitness components of the host, such as resistance to pathogens (reviewed in [14]). In parasitoid wasps, *Wolbachia* has been shown to impose physiological cost to the host (as in *Leptopilina heterotoma* [15]) and to manipulate host reproduction, such as thelytokous parthenogenesis in *Trichogramma* [16, 17] and *Asobara japonica* [12] or cytoplasmic incompatibility in the wasps *Leptopilina* [18] and *Nasonia* [19]. In contrast, some *Asobara* wasps have a strong mutualistic relationship with their *Wolbachia*, which plays an essential role in the development of the host ovaries [20]. In many cases, exactly how *Wolbachia* affects host biology and persists in the host population is unknown.

Prevalence of *Wolbachia* infection varies from 100% to extremely low between host species and among populations within a host species [21, 22]. This range of prevalence comes about because host-symbiont associations either occur under different environmental conditions [23–25]; are at different stages of infection history [26]; or are affected by variable genetic factors of either the symbiont or the host [27, 28]. Furthermore, through manipulation of its host reproductive system *Wolbachia* have the potential to persist in their host population despite obvious costs to host fecundity. CI inducing-*Wolbachia* do this by causing total or partial reproductive failure between males and females with a different infection status, providing a reproductive advantage to female hosts, which can produce viable offspring regardless of whether they mate with infected or uninfected males. *Wolbachia* that distort population sex-ratio (male-killing, parthenogenesis and feminization) promote the production and fitness of infected females, ensuring the transmission of infection through generations [5]. Finally, the coexistence of infected and uninfected individuals in a population is possible if there is perfect vertical transmission of the symbiont [29–32].

In the Åland islands in Southwest Finland, the population dynamics of the Glanville fritillary butterfly, *Melitaea cinxia*, and its specialized parasitoid wasp, *Hyposoter horticola*, are closely linked. The butterfly lives as a metapopulation in a landscape consisting of ~4000 small habitat patches (dry meadows), of which 500 to 800 are occupied yearly. The habitat patches are clustered into ~100 semi-independent networks (SINs) [33] with little gene flow of the butterfly between the networks. In contrast, *H*. *horticola* is highly dispersive among local butterfly populations resulting in high gene flow and low isolation by distance [34]. *Hyposoter horticola* is itself parasitized by a similarly dispersive specialist hyperparasitoid, *Mesochorus* c.f. *stigmaticus* [35]. Of the three species, *H*. *horticola* is the only one yet found to be infected with *Wolbachia* (Duplouy pers. obs.). In such a spatially and trophically structured system, *Wolbachia* epidemiology depends both on the effects of the bacterium on the wasp host, as well as on the demography and population dynamics of its host.

Here, we report three new *Wolbachia* strains, and describe one, *w*Hho, which is present in the Åland islands, at intermediate prevalence. In an attempt to explain the intermediate level of infection, we measured the rate of vertical transmission and the association of infection with several life-history traits. We report spatial associations suggesting that *Wolbachia* infection may influence the resistance of *H*. *horticola* to its common larval specialist hyperparasitoid *M*. c.f. *stigmaticus*.

## Materials and Methods

### Insect material

*Hyposoter horticola* (Gravenhorst)(Hymenoptera: Ichneumonidae: Campoplaginae) is a solitary egg-larval parasitoid of the Glanville fritillary butterfly, *Melitaea cinxia* (L.)(Lepidoptera: Nymphalidae) [36]. The wasp parasitizes about a third of the host caterpillars in the Åland islands [37]. We used *H*. *horticola* reared from naturally parasitized host caterpillars that were systematically sampled in the Åland islands (60°07’N, 19°54’E) between 2008 and 2013 as a part of the long-term ecological study of the Glanville fritillary [33]. To document *Wolbachia* infection over a longer period, we also screened individuals sampled less systematically between 1993 and 2005 and preserved frozen (-20°C) in ethanol. Moreover, to place the samples in a geographical context, we analysed the infection status of *H*. *horticola* collected from Estonia (58°41’N, 22°50’E & 59°35’N, 24°05’E), France (43°61’N, 3°87’E), Spain (37°38’N, 5°98’E) and Sweden (60°00’N, 18°00’E & 56°73’N, 16°67’E). Altogether 751 wasps were screened (Table 1, S1–S3 Tables). The butterfly and its parasitoid are not classified as threatened species and hence no permits are required for their collection in Europe.

**Table 1:** *Wolbachia* infection rate of the two mitotypes of *Hyposoter horticola* in samples collected from Finland, Sweden and Estonia between 1993 and 2013 (for details see S1 and S2 Tables).

Population	*w*Hho-infected		Uninfected	
	C	T	C	T
Estonia	-	-	43	-
Finland (Åland)	75	128	185	27
Sweden	9	15	11	-

### *Wolbachia* strains and *Hyposoter horticola* mitotype diversity

DNA was extracted from the wasp abdomen using a Qiagen DNeasy blood and tissues extraction kit, following the manufacturer’s protocol (Qiagen, USA). The DNA quality was tested by PCR amplifying the mitochondrial *COI* gene (primer pair LCO/HCO [38]). Any sample with no amplification for *COI* was removed. The *COI* locus was sequenced from 514 samples (422, 45, 6, 6 and 35 from Finland, Estonia, France, Spain and Sweden, respectively, S2 Table). *Wolbachia* infection was assessed through the amplification of two to seven *Wolbachia* loci, including the *wsp* gene (primer pair 81F/691R [39]), the *16S-RNA* gene (553F_W/1334R_W [40]) and the five MLST loci (MultiLocus Sequence Typing genes: CoxA, FbpA, HCpA, GatB and FtSZ using respective primers from [41]). We also sequenced two to seven *Wolbachia* loci (MLST, *wsp* and *16s* loci) from 249 (218, 5 and 26 from Finland, France and Sweden, respectively) of the 340 wasps infected with *Wolbachia*. The Fst values, based on the COI sequences, were calculated using Arlequin 3 [42] (S5 Table). Each consensus sequence was submitted to GenBank (KF722987–94, KJ150624, KM598264–68 & KR185318–21).

### Phylogenies

**Fig 1:**
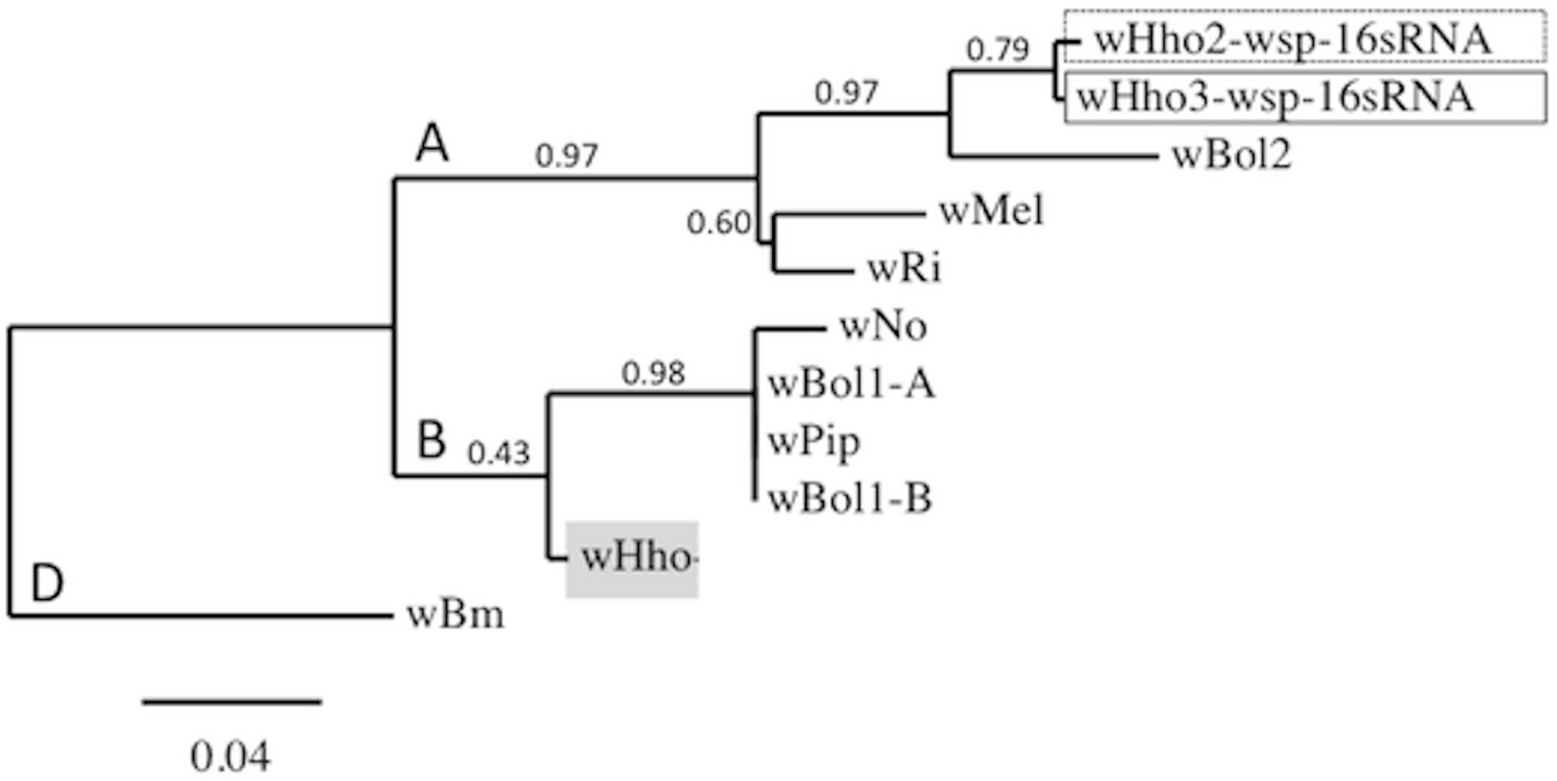
Rooted phylogram based on the concatenated sequences of the *wsp*, *MLST* (*CoxA*, *FbpA*, *FtsZ*, *GatB* and *HcpA*) and *16S* genes from nine *Wolbachia* strains, including *w*Hho, and on the concatenated sequences of *wsp* and *16S* of *w*Ho2 and *w*Hho3, with branch support values. The *w*Bm strain from the parasitic nematode *Brugia malayi* was used as an outgroup. A, B and D are three *Wolbachia* super-groups.

Phylogenies (Fig 1, S1 Fig and S1 File) were built using the online tree-building program *Phylogeny*.*fr* [43, 44] in the *One-click* mode with default settings. For *Wolbachia*, we used the concatenated *wsp*, *16S* and MLST consensus sequences from our three strains and eight additional *Wolbachia* strains (GenBank AM999887, CP001391, CP003883, AE017321, AE017196, EF025179 to -183, EF078895, AB474245 to -249, AB094382, AB052745). We rooted the tree using *w*Bm as an outgroup. For *H*. *horticola* mtDNA, we used the seven mitotypes obtained from our samples together with sequences from five other species of *Hyposoter* (GenBank JF963449, JQ576453, JQ576979 & DQ538855 to -58) and two *Campoletis sonorensis* (Ichneumonidae, GenBank DQ538849 & -50). We rooted the tree using two *Cotesia melitaearum* (Braconidae, GenBank KM598269 & -70) as outgroups.

### Mapping of the infection in the Åland island population

Each wasp was collected from a known geographic location, allowing us to analyse *Wolbachia* prevalence at the levels of local butterfly populations, semi-independent patch network (SIN), and in the landscape as a whole. We used log-linear models to investigate temporal variation in prevalence from 2008 until 2012 in the pooled material for the Åland islands (variables: year & infected versus uninfected) and variation in prevalence among the five SINs with the largest sample sizes (variables: SIN & infected versus uninfected). Finally, we compared infection levels between Sweden and the Åland islands using Fisher’s exact test using pooled data from 2008 to 2012 (categories compared were infected and uninfected individuals from Sweden and Finland).

### *Wolbachia* transmission rate

The vertical transmission of *Wolbachia* is not always perfect [32]. We measured the proportion of *Wolbachia*-infected progeny from 15 female wasps from the Åland islands, eight infected and seven uninfected. Each female parasitized a butterfly host egg-clutch in the laboratory (see [45] for methods). The caterpillars were reared until the 4th instar (15 caterpillars per female) or until wasps emerged as adults. We detected parasitism in the 4th instar caterpillars by amplifying both the parasitoid wasp DNA (microsatellite region Hho10 [46]) and a section of the repeat element protein-b11 from the wasp-integrated ichnovirus (length: 429bp, PCR annealing temperature: 50°C, primers: 34F 5’-GGCGTATGCARTAGTGRTGAA and 463R 5’-GATTATCGGAACCTGRTYGAA, this article). Additionally, because most *H*. *horticola* emerging from the same family group of Glanville fritillary larvae are full siblings [47], we estimated transmission rate in a larger sample using the infection status of wasps emerging from samples of two or three host individual taken from each of 51 host family groups during the field survey (S4 Table). The rate of transmission associated with the two wasp mitotypes was compared using a Fisher’s exact test.

### *Hyposoter horticola* sex ratio

In some systems, *Wolbachia* skews the sex ratio of the host population toward females (through killing or feminization of males, or via parthenogenesis) [5]. As each parasitized *M*. *cinxia* larval group contains primarily offspring of a single *H*. *horticola* female [47], we determined the per-mother allocation to male and female progeny, and the population-level sex ratio, by recording the sex of each individual reared from 53 larval groups parasitized naturally in the Åland islands between 2007 and 2010. Their infection-status is not known. However yearly random collections show that half of all collected wasps carry *w*Hho in the Åland islands (S1 Table), so we assume that around half of these 53 larval groups originate from infected wasps. The mean brood sex ratio (fraction male) was compared with an equal (0.5) sex ratio using t-test. The distribution of brood sex ratios was compared with a normal distribution using the Shapiro-Wilk goodness of fit test.

### *Hyposoter horticola* egg-load

*Wolbachia* is known to affect host fecundity [7, 9]. We tested for an association between the infection status and egg-load (as proxy for fecundity) of 213 females (including 111 infected and 102 uninfected) ranging from age 0 (day of emergence) to 51 days (maximum lifespan in the laboratory). The wasps were reared from naturally parasitized caterpillars and randomly killed at different ages, yielding a similar sample of infected and uninfected individuals at each age point. The ovaries were dissected in sterile conditions and mature eggs in the oviduct and calyx organs were counted as part of another study [45]. The infection status of each wasp was determined upon death. The egg load was then analysed using a linear model, with the infection status and age of the wasp as explanatory variables.

### *Hyposoter horticola* longevity

Presence of *Wolbachia* can decrease host longevity [11]. Fifteen females and 33 males, reared under laboratory conditions from caterpillars naturally parasitized in the Åland islands in 2010 and 2011, were kept in individual vials (V = 100 ml) in an incubator at 12:12 L/D and 18/10°C day/night temperature. Wasps were fed 30% honey solution every other day until natural death (as described in [45]), and later assessed for *Wolbachia* infection. We tested the association of longevity with infection status using a linear model, with sex included as an extra explanatory variable.

### Association of *Wolbachia* infection with habitat connectivity

If *Wolbachia* infection decreases dispersal ability of wasps, we would expect low prevalence of the bacterium where local butterfly populations are sparse in the landscape (low connectivity) [48], as the chances for *Wolbachia*-infected wasps to reach the surrounding habitat patches would be reduced. Alternatively, if the bacterium increased dispersal ability [49], we might expect prevalence to be lower in highly connected habitat patches. For each year (2008–2013), we calculated an index of connectivity of each local butterfly population from which wasps were reared, accounting for the distance from each local butterfly population to all other populations, the sizes of the populations and the dispersal ability of the butterfly [50, 51]. Using a linear model we tested possible association of the infection rate of *H*. *horticola* in a local butterfly population in year *t* with the connectivity of the population in year *t*-1, as wasps collected in the current year were the progeny of adults from the previous summer.

### *Wolbachia* infection and host metabolic rate

*Wolbachia* can influence basic physiological and metabolic functions of its host. This would be especially relevant for the dispersal ability of the wasp, as flight is metabolically expensive [52, 53]. We compared the active and resting metabolic rates (AMR and RMR, respectively) of 33 *Wolbachia*-infected and 29 uninfected wasps reared from caterpillars collected from the wild in 2013. The metabolic rates were measured using flow-through respirometry at the constant temperature of 30°C [53]. The AMR was measured as the maximum rate of CO_2_ emission under a bright light during 7 min of forced activity induced by flicking the 100 ml metabolic chamber each time the wasp’s wings were still (*H*. *horticola* may fly for more than 30 min without exhaustion, Duplouy, pers. obs.). We used the average CO_2_ emission rate during 60 sec preceding the active period, under a cover to block light, as a measure of the of the baseline RMR. Measures of AMR and RMR were corrected for variation in body mass by using residuals from linear regression between the metabolic rate and the weight. Linear models were used to analyse associations of CO_2_ emission rate with the infection status and sex of individuals.

### Prevalence of *Wolbachia* and the rate of hyperparasitism

If *Wolbachia* confers resistance against parasitoids, we might expect the bacterial infection to be high where the risk of parasitism is high [54]. We used linear regression to compare the prevalence of *Wolbachia* infection in *H*. *horticola* and the rate of hyperparasitism by *Mesochorus* c.f. *stigmaticus* using data from ten semi-independent patch networks (SINs) in the Åland islands. For each SIN, we calculated the relative abundance of the hyperparasitoid as the number of hyperparasitoids in the sample divided by the sum of the parasitoids and hyperparasitoids.

### Data access and analyses software

Data files are available in *Dryad* under the doi: 10.5061/dryad.34sv3. Statistical analyses were performed using R64 [55].

## Results

### *Wolbachia* infection in *Hyposoter horticola*

The Finnish and Swedish populations of *H*. *horticola* carry a common *Wolbachia* strain that belongs to the *Wolbachia* B-supergroup (Fig 1), and which we name *w*Hho (ST435). A single specimen from the Åland islands (sampled in 1998) was infected by an A-supergroup strain, *w*Hho3. Samples from Estonia (*n* = 43) and Spain (*n* = 6) were uninfected, while samples from France (*n* = 6) carried a third strain, called *w*Hho2, also in the A-supergroup.

**Fig 2:**
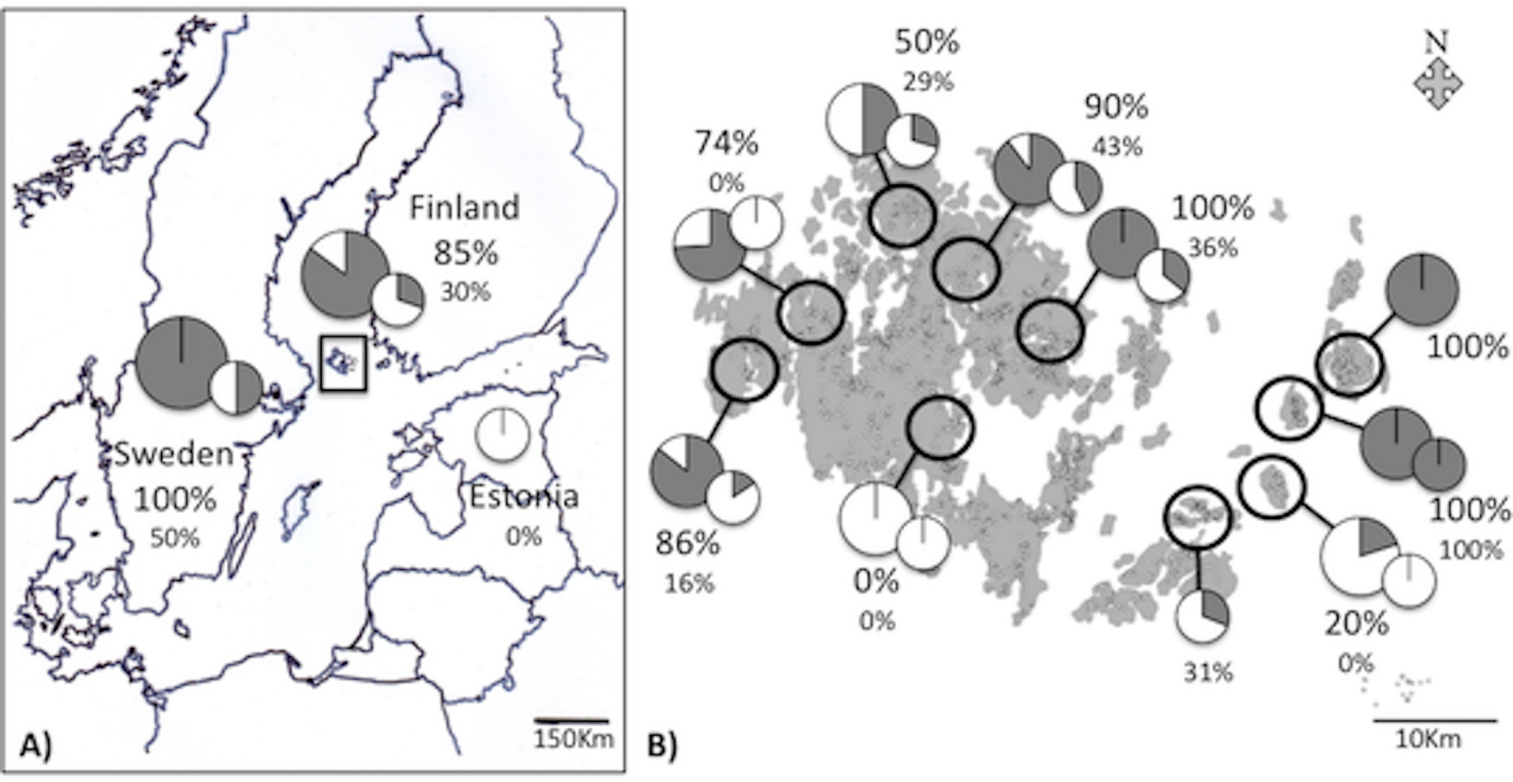
Geographical pattern of *Wolbachia* infection rate (in grey) in samples at the (A) regional scale in Finland, Sweden and Estonia, and (B) among networks of habitat patches in the Åland islands in Finland. The data includes samples collected between 2008 and 2013. Large circles show the infection rate in the T mitotype and small circles in the C mitotype. When one mitotype is absent, the respective circle is also absent (e.g. only the C mitotype occurs in Estonia).

**Fig 3:**
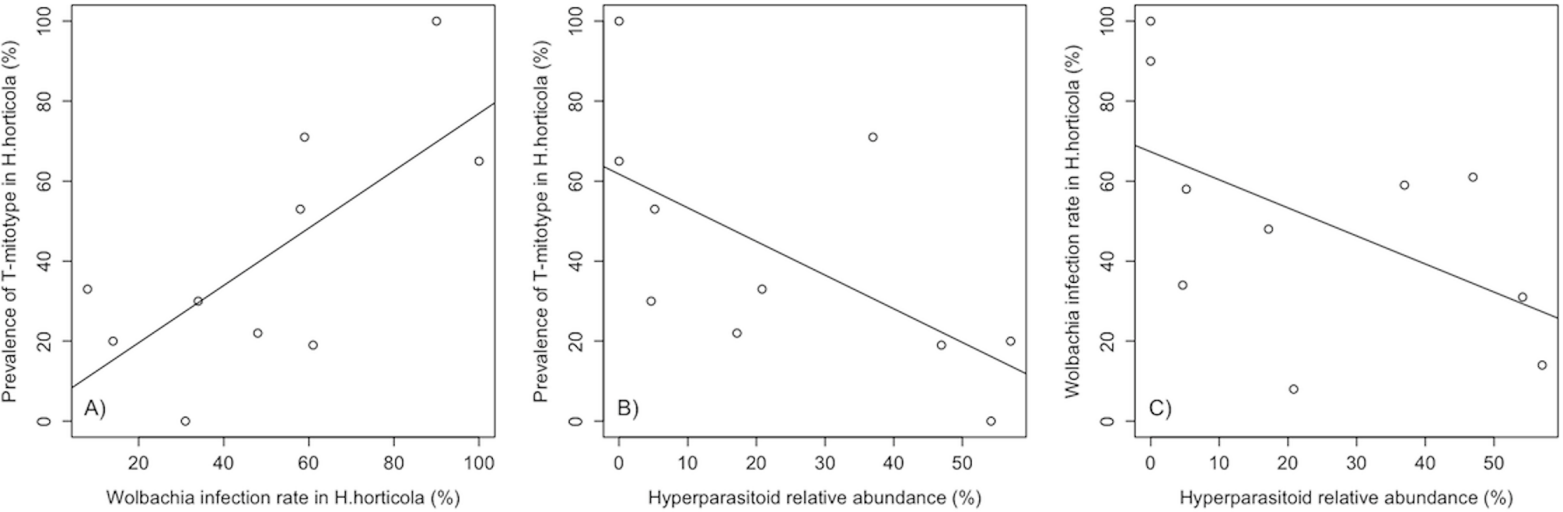
Relationships between *Hyposoter horticola* mitotype, *Wolbachia* infection and hyperparasitism by *Mesochorus* c.f. *stigmaticus* in 10 sub-regions in the Åland islands. The data were collected from 2008 to 2013. Panel (A) shows the association between *Wolbachia* infection rate and the relative abundance of the T mitotype in *H*. *horticola* (*P* = 0.01). The next two panels show the relationships between the relative abundance of the hyperparasitoid and (B) the relative abundance of the T mitotype (*P* = 0.039) and (C) *Wolbachia* infection rate (*P* = 0.11).

In the Åland islands and Sweden, *Wolbachia* infection rate is related to the mitochondrial haplotype (mitotype) of the host. Based on the *COI* sequence, there are two common mitotypes, which differ by a single base pair substitution (synonymous substitution between C and T at the nucleotide position 364, the codon encoding for Leucine at position 121). Individuals with mitotype T are more likely to be infected by *Wolbachia* than individuals with mitotype C (Åland: 87% vs. 30%; Sweden: 100% vs. 56%, Fisher’s exact tests *P* = 0.0001 and *P* = 0.0068, respectively, Fig 2). The overall infection rate is significantly higher in samples from Sweden than from the Åland islands (*P* = 0.012). Within the Åland islands, from where we have samples from 2008 until 2013, the overall infection rate has remained stable (non-significant interaction between year and infection rate, df = 4, *P* = 0.21). However, there are highly significant differences in the infection rate between SINs (df = 16, *P<*0.001, Fig 2), and the overall infection rate is correlated with the frequencies of the two mitotypes (df = 1, *P* = 0.039, Fig 3).

### Transmission rate

Of the fifteen female wasps used in the transmission experiment, eight were infected by *Wolbachia*. Five of the eight showed high *Wolbachia* transmission (>85%), while the remaining three showed lower transmission (between 33 and 66%). None of the offspring of the seven uninfected mothers were infected (S4 Table). As only one of the eight infected females had mitotype C, this material cannot be used on its own to compare the two mitotypes. We therefore augmented the lab-reared material with the field-collected samples. Among the presumed sibling groups produced by an infected individual of the C mitotype, nine out of 17 (53%) included one uninfected offspring (two to seven offspring per mother). In contrast, among the presumed siblings groups from infected females with mitotype T, only five out of 30 (17%) sibling groups had at least one uninfected individual. These results suggest that transmission is less efficient in the C mitotype (Fisher’s exact test, *P* = 0.018). The absolute values of uninfected individuals are likely to be biased, as some infected females of either mitotype may have had only uninfected offspring, and hence the female was wrongly scored as uninfected. However, comparison of the two mitotypes suggests more efficient transmission in the T mitotype.

### Life-history and fitness consequences of *Wolbachia* infection

In the Åland islands, *H*. *horticola* has an equal population-level sex ratio (0.54, SD = 0.24), which does not differ from 0.5 (*t* = 1.01, *P*>0.05). Moreover, the brood level sex ratio was normally distributed around the mean in wasps from 53 host larval family groups (Shapiro-Wilk Goodness-of-fit test, W = 0.9827, *P* = 0.78). There is thus no evidence for extreme sex ratio distortion. However, these results do not rule out the possibility of other reproductive manipulations apart from sex ratio distortion, such as CI.

**Fig 4:**
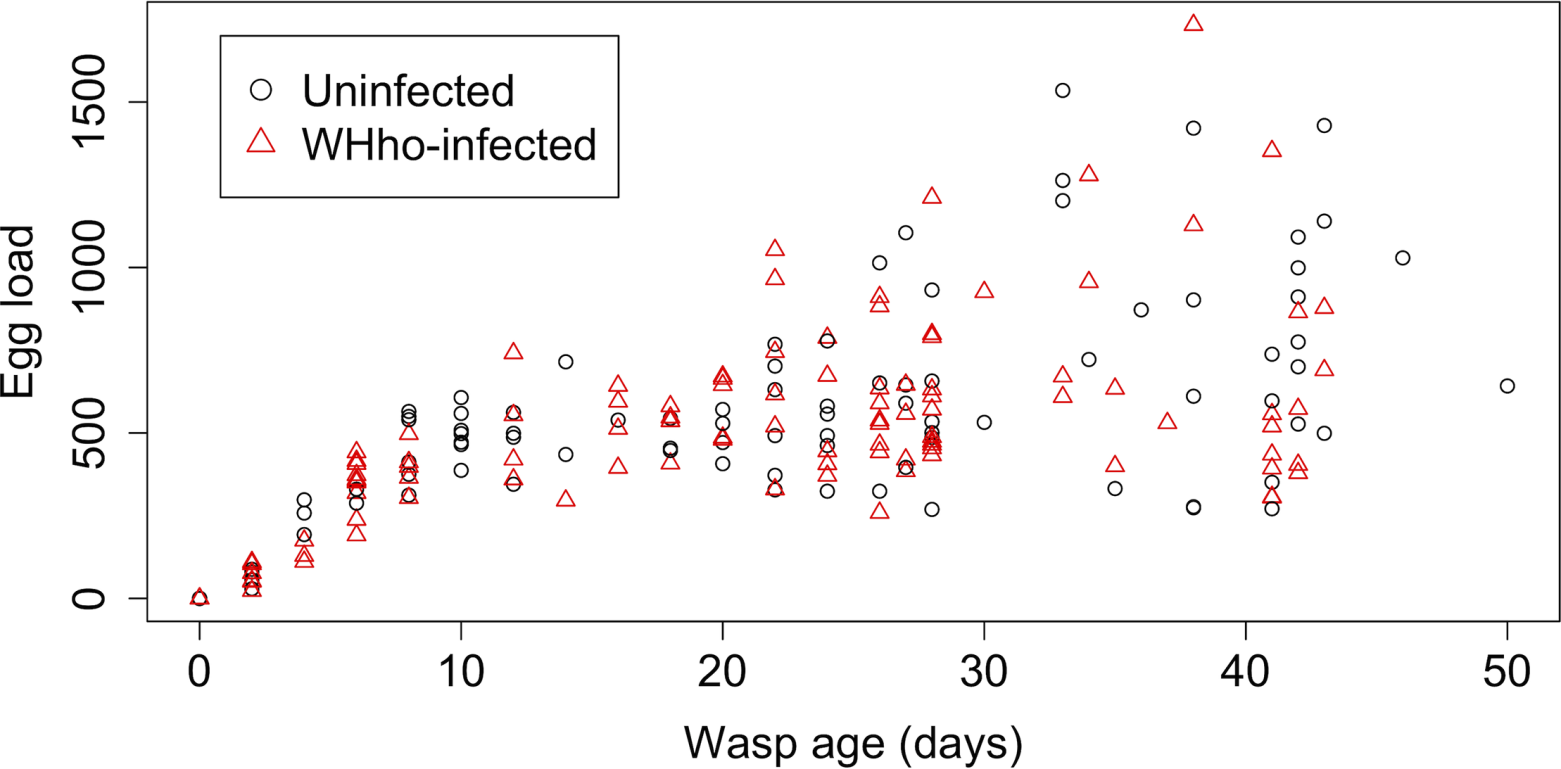
The egg-load of female wasps from the Åland islands in relation to their age and whether they are infected by *Wolbachia* (triangles, *n* = 111) or not (circles, *n* = 102).

We found no association of *Wolbachia* infection with fitness-related traits in *H*. *horticola*. Infected and uninfected females had similar egg-load (520 and 522, df = 1, *P*>0.05, Fig 4). Females lived longer than males (37 versus 31 days, df = 1, *P* = 0.036), and *Wolbachia* infection was unrelated to longevity in either sex (df = 1, *P*>0.05, S2 Fig).

We found no association of *Wolbachia* infection with habitat connectivity in the previous year (Fig A in S2 File), suggesting that the infection does not influence wasp mobility. Consistent with this negative result, there was no association of *Wolbachia* infection with active or resting *metabolic* rate (Figs B and C in S2 File).

### Parasitoid-hyperparasitoid interaction and *Wolbachia* infection

We analysed the relative abundances of *H*. *horticola* and its hyperparasitoid *Mesochorus* c.f. *stigmaticus*, as well as *Wolbachia* infection rate, in ten regions in the Åland islands (Fig 2B). The hyperparasitoid had greater relative abundance where the prevalence of the host T mitotype was lower (*P =* 0.039). Recalling that *Wolbachia* infection rate is higher among T- than C-mitotype wasps (above), this result suggests the hypothesis that *w*Hho infection protects *H*. *horticola* against the hyperparasitoid. Indeed, in these data *Wolbachia* infection rate is low where hyperparasitism is high, though the association is not statistically significant (*P* = 0.11, Fig 3C). In Estonia, where *Wolbachia* is entirely absent, all the wasps are of mitotype C and the hyperparasitoid is absent. Under the hypothesis that *Wolbachia* protects against hyperparasitism, we would expect *Wolbachia* to be absent where there is no hyperparasitoid because, as it would be no advantage to its host, it would not spread.

## Discussion

We describe three *Wolbachia* strains in the ichneumonid wasp *Hyposoter horticola*, a parasitoid of the Glanville fritillary butterfly: two A-group strains, *w*Hho2 and *w*Hho3, detected from French samples and a unique Finnish sample, respectively, and a B-group strain, *w*Hho (ST435), which is widespread in the Åland islands in Finland and in Sweden. No *Wolbachia* was identified in samples from Estonia (*n* = 47). The host *H*. *horticola* has seven *COI* mitotypes in our material, of which the two common ones (mitotypes C and T) occur in Finland and Sweden, one rare mitotype occurs in Estonia, and four other divergent mitotypes came from France and Spain. The post-glacial range expansion of *H*. *horticola* is unclear, but it may have spread to northern Europe, possibly with different *Wolbachia* strains, from both Asian and southern European refugia, as has its host butterfly [56] and another specialist parasitoid *Cotesia melitaearum* [57].

### Rate of transmission and association of *Wolbachia* infection with host mitotype

We found that in the Åland islands the rate of vertical transmission of *Wolbachia* is imperfect, suggesting that the infection persists in an intermediate state, in balance between fitness benefits and costs [58, 59]. Moreover, the two *H*. *horticola* mitotypes are not equally infected. In the Finnish and Swedish populations, *Wolbachia* is common in the host mitotype T (92%) but much less common in the C mitotype (43%). In Estonia, all wasps had the C mitotype and were uninfected.

Both mitochondria and *Wolbachia* are transmitted maternally. Hence patterns of mtDNA-*Wolbachia* association reflect infection biogeography, strength of transmission, and history of *Wolbachia* spread in host populations [60, 61]. When vertical transmission is efficient, *Wolbachia* infection may result in a selective sweep leading to distinct infected and uninfected matrilines ([26, 62] e.g. in butterflies [63] and fig wasps [64]). When transmission is imperfect or when there is horizontal transfer, infected and uninfected individuals may share the same mitotypes [65]. Infection rate of the *w*Hho strain in Finland and Sweden may be higher in the T than the C mitotype because *w*Hho infected the common ancestral matriline of both mitotypes, but transmission rate is higher in the T mitotype. Alternatively, the *w*Hho strain may have first arisen in the T mitotype and later colonized the C mitotype by lateral transfer. Such lateral transmission could occur if two individuals parasitize the same host (superparasitism), which can occasionally happen in *H*. *horticola* [37]. Alternatively the hyperparasitoid *M*. c.f. *stigmaticus* could transmit the bacterium between hosts (e.g. in aphids [66]). Finally, it is also possible that the mitotype diversity is higher than what the *COI* gene alone indicates, which would increase the complexity of the system.

### *Wolbachia* infection and host fitness

*Wolbachia* is known to influence host fitness, reproductive system and behaviour to improve its transmission [67]. In this study the prevalence of *w*Hho differed geographically between the study regions, from 0 to 74%, at the scale of several hundred km (Fig 2A), as well as at a smaller spatial scale within the Åland islands, where infection rate varies from 8 to 100% regionally (Fig 2B). With efficient transmission, as appears to happen in the T mitotype, the bacterium would need to enhance host fitness only little, or to induce a strong CI, to persist. In the C mitotype with less efficient vertical transmission, the fitness benefit would need to be greater to compensate for the lower transmission rate.

Models predict that high variation in prevalence among populations may occur when there is high spatial population dynamics [59, 68]. Environmental heterogeneity in the Åland islands leading to large variation and fluctuation of local host population sizes [69] and prevalence of the hyperparasitoid *M*. c.f. *stigmaticus* [35], may interact with the effects of *Wolbachia* on its host, leading to spatial variation in prevalence.

Longevity and fecundity are correlated with fitness in parasitoids [70], and are often affected by *Wolbachia* [15, 71]. *Wolbachia* infection can also influence behavioural traits, such as dispersal in mosquitos [49]. However, we found no evidence for *w*Hho influencing host wasp longevity, fecundity or dispersal in the field. The lack of an association of infection with life history or behaviour suggests that the pattern of *Wolbachia* infection in the Åland islands may simply be in a transitional state, on its way toward fixation or extinction (e.g. in butterflies [26]). However, the spread of a CI-inducing or a beneficial strain could also show such heterogeneity between local populations [72, 73].

Alternatively, *Wolbachia* may have a less commonly studied association with fitness. One interesting hypothesis, suggested by our results, is that *Wolbachia* confers resistance to hyperparasitism. Several symbionts, including *Wolbachia* ([74] but see [75, 76]) have been found to protect their hosts against parasitoids (e.g. in aphids [77, 78] and fruit flies [79]). The specialist hyperparasitoid *M*. c.f. *stigmaticus* is ubiquitous in the Åland islands and Sweden [35], where *Wolbachia* prevalence is greater than 50%, while *M*. c.f. *stigmaticus* is absent from Estonia [37], where *Wolbachia* is also absent. Furthermore, within the Åland islands, the relative abundance of the hyperparasitoid decreased with increasing prevalence of the host T mitotype. As the T mitotype is also more often associated with *Wolbachia* than the C mitotype, we suggest that the presence of *Wolbachia* may improve resistance to hyperparasitism. Thus, *w*Hho could be maintained because the infection reduces the likelihood of successful hyperparasitism.

To conclude, in this comprehensive landscape-level study of *Wolbachia* prevalence in a large spatially structured wasp population, there is a stable intermediate *Wolbachia* infection rate, which is significantly different in two host mitochondrial haplotypes (mitotypes). This pattern has several possible explanations, including imperfect but dissimilar vertical transmission rate in the two mitotypes. Second, we found no direct effect of infection, positive or negative, on fitness-related traits of the host, including egg-load (fecundity), adult longevity, metabolic rate and a measure reflecting dispersal rate in the field. Third, we have no evidence of sex-ratio distortion in the host, though we cannot rule out the expression of CI and its consequences for the spread of the bacterium in the population. The lack of a positive association with host fitness may indicate that *Wolbachia* is in a transitional state in the landscape, or that it is maintained via some other mechanism. One such mechanism is protection against hyperparasitism. We do not have direct experimental evidence for this hypothesis, but it is supported by the distributional data showing association of infection with the occurrence of the hyperparasitoid across the study landscape.

## Supporting Information

S1 Fig Rooted phylogram based on the *COI* gene sequences from several *Hyposoter* species including *H*. *horticola*, and two specimens of a second ichneumonidae wasp, *Campoletis sonorensis*. *COI* sequences of two specimens of the Braconidae *Cotesia melitaearum* were used as outgroups. The mitotypes associated with *w*Hho are shown in grey, and those associated with *w*Hho2 are encircled by a dashed-line.
(TIFF)

S2 Fig Longevity of *w*Hho-infected (grey, N_wHho_ = 27) and uninfected (white, N_U_ = 21) male and female *Hyposoter horticola* from the Åland islands. (TIFF)

S1 File Rooted phylograms based on the sequences of (Fig A) wsp, (Fig B) 16sRNA and (Figs C-G) each MLST gene from several *Wolbachia* strains, including *w*Hho. (Figs A and B): The *w*Hho2 and *w*Hho3 are only included in the trees built on *wsp* and *16s* gene sequences, respectively. The *w*Bm strain is used as an outgroup in each phylogeny. The phylogenies resemble each other and the phylogeny constructed with the concatenated sequence of each gene (Fig 1), suggesting absence of recombination.
(TIFF)

S2 File (Fig A) Presence/absence of *Wolbachia* in *Hyposoter horticola* samples from local host butterfly populations in the Åland islands between 2008 and 2013 (time t), and the mean butterfly population connectivity in the previous year (t-1). Connectivity differs significantly between the years (*P* = 7.06e-7), but is not related to *Wolbachia* infection status. Weight-corrected (Fig B) active and (Fig C) resting metabolic rates of *H*. *horticola* infected or not with *Wolbachia*.
(TIFF)

S1 Table Sample size and *w*Hho infection status for each year. (*) indicates samples infected by a less commonly found *Wolbachia* strain, *w*Hho2 or *w*Hho3. SA: Estonian island of Saaremaa, VÄ: mainland Sweden, Väddö, ÖL: Swedish island of Öland.
(DOCX)

S2 Table Year-specific *Wolbachia* infection status and associated *Hyposoter horticola* mitotype. (DOCX)

S3 Table Sample sizes for the studies. Sample size in the transmission experiment is presented in S4 Table. The mapping study includes an additional 28 uninfected samples for which sex is unknown.
(DOCX)

S4 Table Transmission rate of *Wolbachia*, *w*Hho, in the two matrilines of *Hyposoter horticola* collected from the Åland islands. In the laboratory experiment, females with less than 3 offspring were not included in the analysis.
(DOCX)

S5 Table Population pairwise *F*_*st*_ values for *Hyposoter horticola* (below diagonal), average number of pairwise differences within population (π_x_, diagonal bold elements), and average number of pairwise differences between populations (π_xy_, above diagonal). (DOCX)
