# Supplementary figures and images for: Wolbachia Infection in a Natural Parasitoid Wasp Population
Source: PLoS One. 2015 Aug 5;10(8):e0134843. doi: 10.1371/journal.pone.0134843 (PMC4526672; doi:10.1371/journal.pone.0134843)

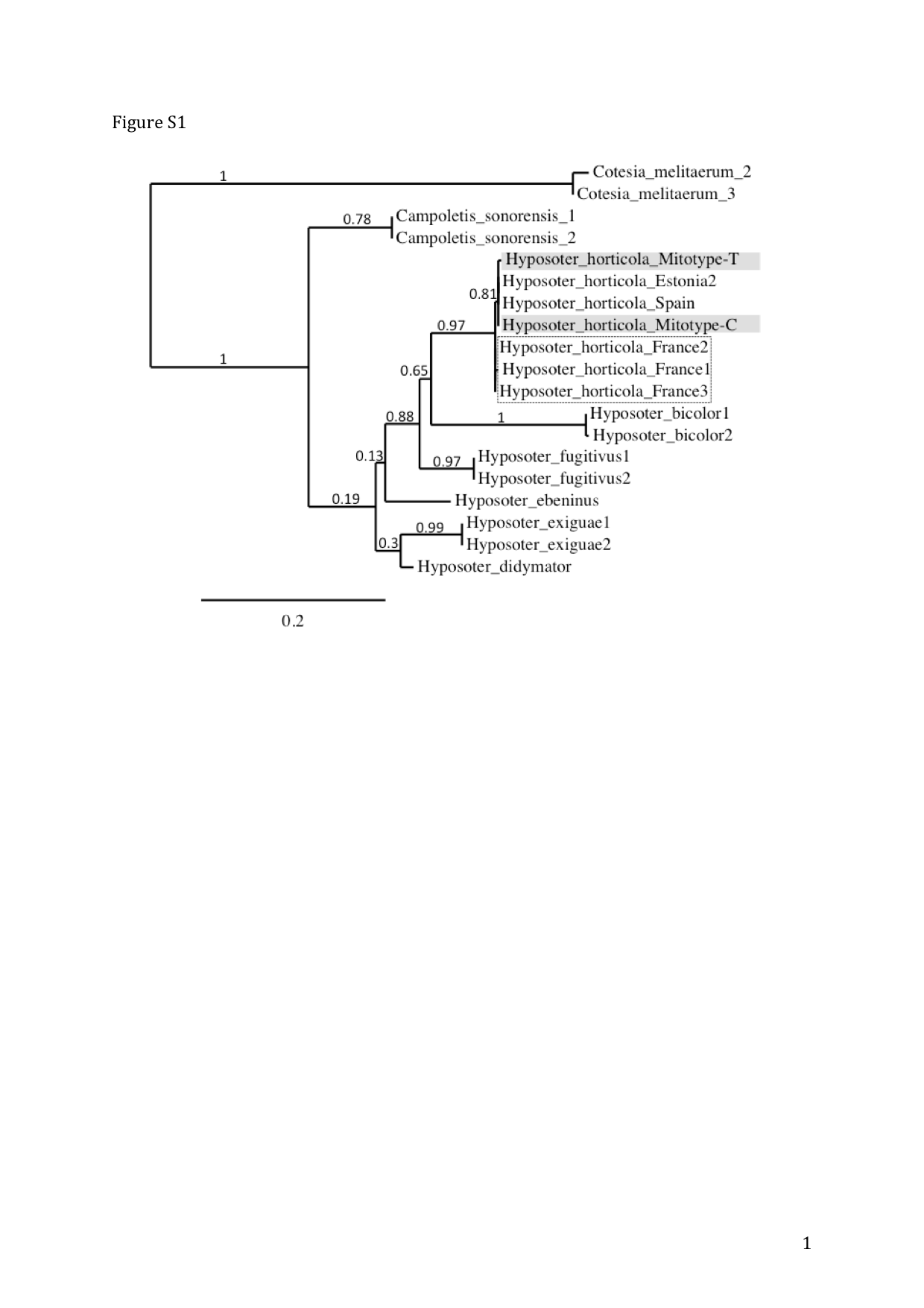

Supplement: S1 Fig — COI sequences of two specimens of the Braconidae Cotesia melitaearum were used as outgroups. The mitotypes associated with wHho are shown in grey, and those associated with wHho2 are encircled by a dashed-line. (TIFF) [file pone.0134843.s001.tiff]

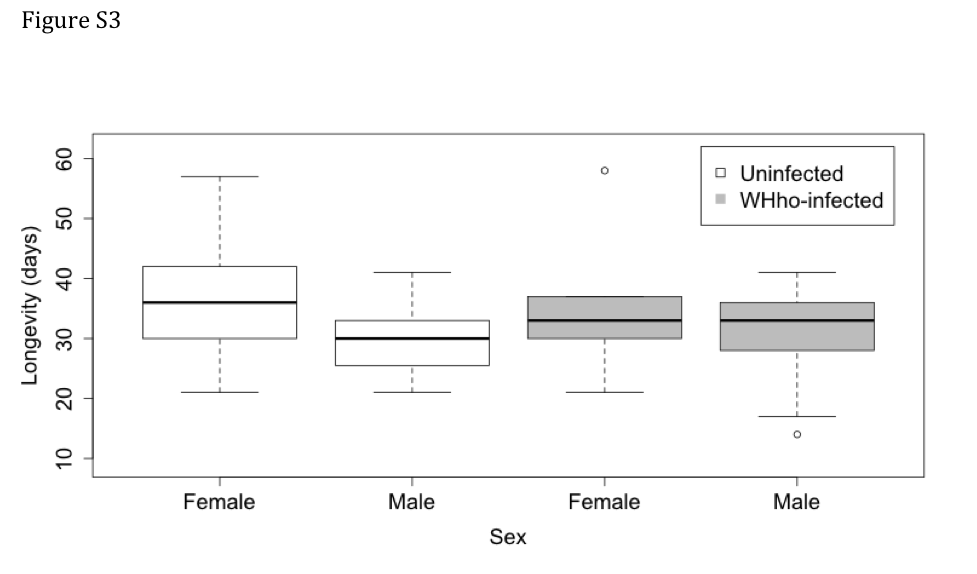

Supplement: S2 Fig — (TIFF) [file pone.0134843.s002.tiff]

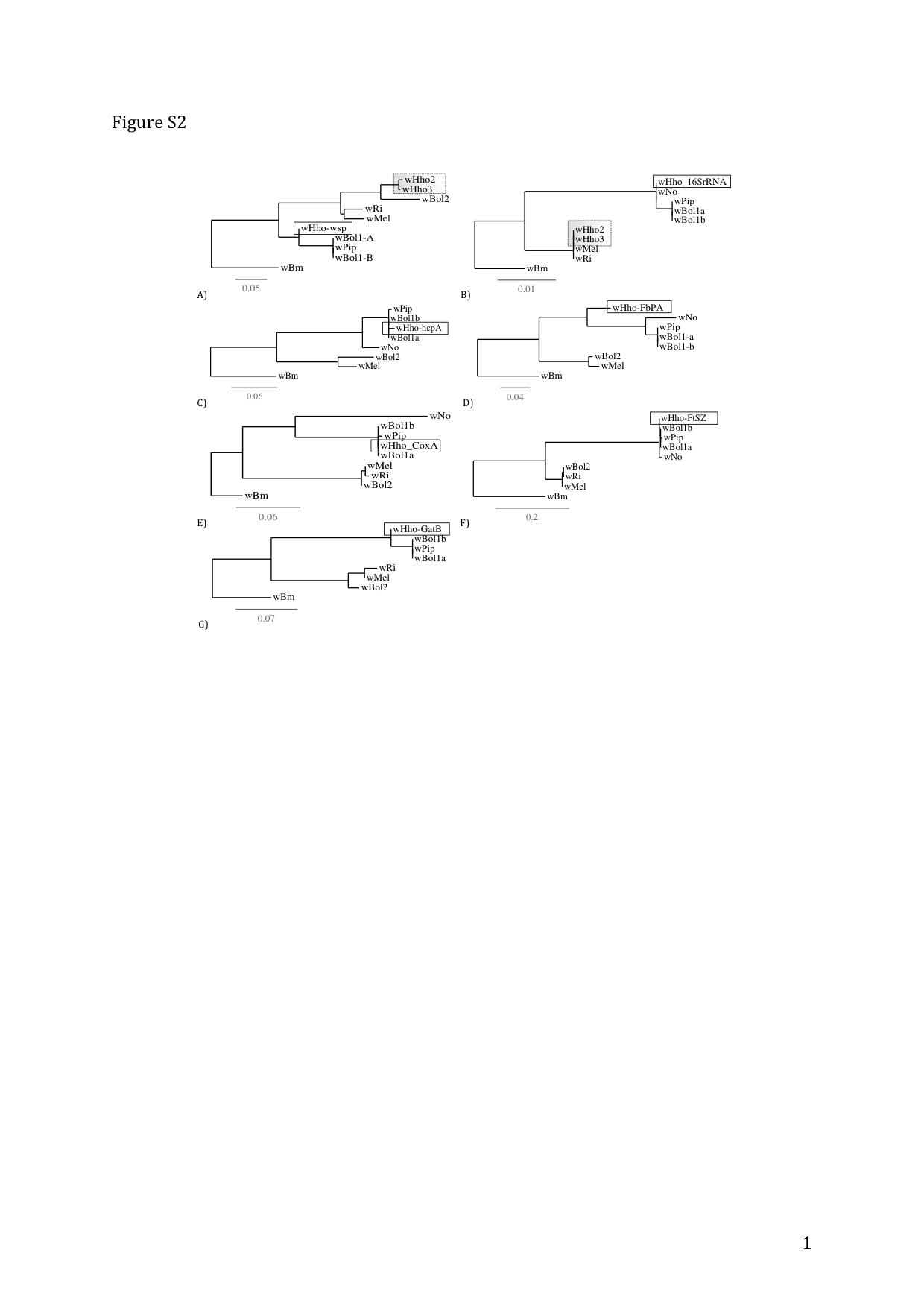

Supplement: S1 File — (Figs A and B): The wHho2 and wHho3 are only included in the trees built on wsp and 16s gene sequences, respectively. The wBm strain is used as an outgroup in each phylogeny. The phylogenies resemble each other and the phylogeny constructed with the concatenated sequence of each gene (Fig 1), suggesting absence of recombination. (TIFF) [file pone.0134843.s003.tiff]

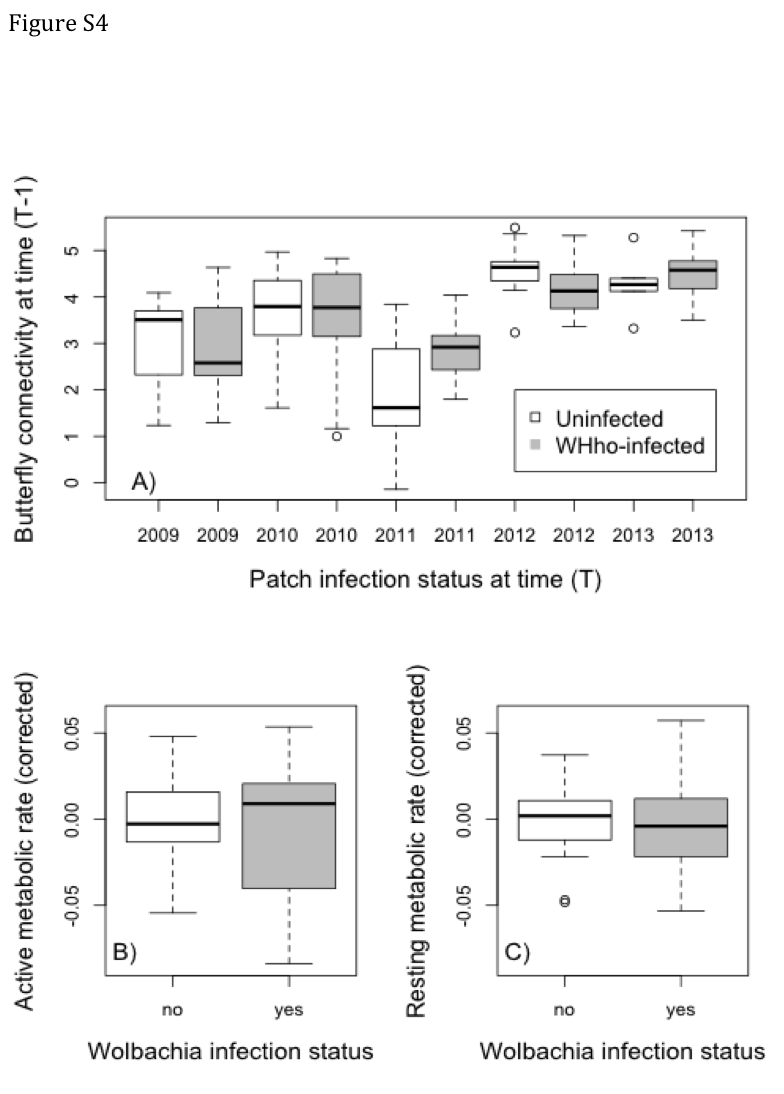

Supplement: S2 File — Connectivity differs significantly between the years (P = 7.06e-7), but is not related to Wolbachia infection status. Weight-corrected (Fig B) active and (Fig C) resting metabolic rates of H. horticola infected or not with Wolbachia. (TIFF) [file pone.0134843.s004.tiff]
