# Supplementary material for: Wolbachia Infection in a Natural Parasitoid Wasp Population
Source: PLoS One. 2015 Aug 5;10(8):e0134843. doi: 10.1371/journal.pone.0134843 (PMC4526672; doi:10.1371/journal.pone.0134843)
Supplement: S1 Table — (*) indicates samples infected by a less commonly found Wolbachia strain, wHho2 or wHho3. SA: Estonian island of Saaremaa, VÄ: mainland Sweden, Väddö, ÖL: Swedish island of Öland. (DOCX) [file pone.0134843.s005.docx]

**Supplementary material- Table S1.**

|  | Sex | | | | |  | |
| --- | --- | --- | --- | --- | --- | --- | --- |
|  | Male | | Female | | Unknown |  |  |
| Year | Infected | Uninfected | Infected | Uninfected | Uninfected | Country | Prevalence |
| 1992 | - | 2 | - | - | - | Finland | - |
| 1993 | - | 1 | - | - | 1 | Finland | - |
| 1997 | -  - | 2  1 | -  1 | 1  1 | -  - | Finland Sweden (ÖL) | -  - |
| 1998 | - | 4 | 1* | 1 | - | Finland | - **^w^*^Hho3^ |
| 1999 | -  - | 7  - | -  - | -  - | 4  4 | Estonia  Finland | 0%  - |
| 2001 | -  1  - | 11  2  - | -  -  - | 10  1  6 | 2  -  - | Estonia (SA)  Finland  Spain | 0%  -  - |
| 2002 | - | - | - | 2 | 9 | Finland | - |
| 2003 | 6 | 11 | 2 | 3 | 1 | Finland | 36% |
| 2004 | - | - | - | - | 6 | Finland | - |
| 2005 | 2 | 1 | 3 | 1 | 4 | Finland | - |
| 2008 | 13 | 19 | 6 | 12 | - | Finland | 38% |
| 2009 | 45 | 41 | 49 | 30 | 3 | Finland | 57% |
| 2010 | 42  -  0  3 | 22  2  3  2 | 69  -  3  19 | 85  -  1  4 | -  -  -  - | Finland  Estonia (SA)  Sweden (VÄ)  Sweden (ÖL) | 51%  -  -  78.5% |
| 2011 | 4  4** | 3  1 | 9  1** | 11  - | -  - | Finland  France | 48%  83%***^w^*^Hho2^ |
| 2012 | 3  - | 3  3 | 18  - | 16  7 | -  - | Finland  Estonia | 52.5%  0% |
| 2013 | 15 | 17 | 19 | 15 | - | Finland | 51.5% |
