# Supplementary material for: Wolbachia Infection in a Natural Parasitoid Wasp Population
Source: PLoS One. 2015 Aug 5;10(8):e0134843. doi: 10.1371/journal.pone.0134843 (PMC4526672; doi:10.1371/journal.pone.0134843)
Supplement: S2 Table — (DOCX) [file pone.0134843.s006.docx]

**Supplementary material- Table S2.**

| Infection | | *w*Hho-infected | | Uninfected | | Alternative association |
| --- | --- | --- | --- | --- | --- | --- |
| Mitotype (*COI*) | | C | T | C | T |  |
| Estonia | 1999 | - | - | 11 | - | - |
|  | 2001 (SA) | - | - | 21 | - | 2 uninfected |
|  | 2010 (SA) | - | - | 2 | - | - |
|  | 2012 | - | - | 9 | - | - |
| Finland (Åland) | 1993 | - | - | 1 | - | - |
|  | 1997 | - | - | 2 | 1 | - |
|  | 1998 | - | - | 4 | - | 1x ”C” *^w^*^Hho3^ |
|  | 1999 | - | - | 2 | 1 | - |
|  | 2001 | 1 | - | 3 | - | - |
|  | 2002 | - | - | 9 | 2 | - |
|  | 2003 | 2 | 4 | 15 | - | - |
|  | 2004 | 2 | - | 5 | 1 |  |
|  | 2005 | 3 | 2 | 5 | 1 | - |
|  | 2008 | 1 | 16 | 23 | 2 | - |
|  | 2009 | 32 | 47 | 51 | 6 | - |
|  | 2010 | 15 | 34 | 29 | 5 | - |
|  | 2011 | 2 | 1 | 2 | 1 | - |
|  | 2012 | 4 | 17 | 12 | 5 | - |
|  | 2013 | 13 | 6 | 22 | 2 |  |
| France | 2011 | - | - | - | - | 6*^w^*^Hho2^ |
| Spain | 2001 | - | - | - | - | 6 uninfected |
| Sweden | 1997 (ÖL) | 1 | - | 1 | - | - |
|  | 2010 (VÄ) | 8 | 15 | 10 | - | - |
