# Supplementary material for: Wolbachia Infection in a Natural Parasitoid Wasp Population
Source: PLoS One. 2015 Aug 5;10(8):e0134843. doi: 10.1371/journal.pone.0134843 (PMC4526672; doi:10.1371/journal.pone.0134843)
Supplement: S3 Table — Sample size in the transmission experiment is presented in S4 Table. The mapping study includes an additional 28 uninfected samples for which sex is unknown. (DOCX) [file pone.0134843.s007.docx]

**Supplementary material- Table S3.**

| Experiment | | Mapping  (2008-2013) | | Longevity | | | Egg-load | |
| --- | --- | --- | --- | --- | --- | --- | --- | --- |
| Infection | Infected | | Ø | Infected | Ø | Infected | | Ø |
| Male | 125 | | 115 | 21 | 12 | - | | - |
| Female | 192 | | 181 | 6 | 9 | 111 | | 102 |
