# Supplementary material for: Wolbachia Infection in a Natural Parasitoid Wasp Population
Source: PLoS One. 2015 Aug 5;10(8):e0134843. doi: 10.1371/journal.pone.0134843 (PMC4526672; doi:10.1371/journal.pone.0134843)
Supplement: S4 Table — In the laboratory experiment, females with less than 3 offspring were not included in the analysis. (DOCX) [file pone.0134843.s008.docx]

**S4 Table**

|  | Female (ID) | Offspring (N=) | Infected offspring (N=) | Transmission rate |
| --- | --- | --- | --- | --- |
| Laboratory transmission experiment | | | | |
| Mitotype C | WA12-029  *WA13-094*  *WA12-121* | 16  *2*  *1* | 14  *2*  *0* | 87,5%  *100%*  *0%* |
| Mitotype T | WA12-140  WA12-030  WA12-315  WA12-061  WA12-174  WA12-336  WA12-354  *WA12-341*  *WA13-388* | 40  6  5  3  3  3  3  *2*  *2* | 37  4  5  1  2  3  3  *0*  *2* | 92,5%  66,7%  100%  33,3%  66,7%  100%  100%  *0%*  *100%* |
| Uninfected controls | 7 females | 48 | 0 | 0% |
| Sibling groups from the field | | | | |
| Mitotype C | (N=16) | 1x 7siblings  2x 3siblings  1x 3siblings  7x 2siblings  5x 2siblings | 1x 7siblings  2x 2siblings  1x 1sibling  7x 2siblings  5x 1sibling | 100%  2x 66%  1x 33%  7x 100%  5x 50% |
| Mitotype T | (N=23) | 3x 3siblings  1x 3siblings  18x 2siblings  1x 2siblings | 3x 3siblings  1x 1sibling  18x 2siblings  1x 1sibling | 3x 100%  1x 33%  18x 100%  1x 50% |
| Uninfected controls | (N=32) | 1x 5siblings  2x 4siblings  5x 3siblings  22x 2siblings | 0  0  0  0 | 0%  0%  0%  0% |
