# Supplementary material for: Wolbachia Infection in a Natural Parasitoid Wasp Population
Source: PLoS One. 2015 Aug 5;10(8):e0134843. doi: 10.1371/journal.pone.0134843 (PMC4526672; doi:10.1371/journal.pone.0134843)
Supplement: S5 Table — (DOCX) [file pone.0134843.s009.docx]

**S5 Table**

|  | Estonia | Åland | Sweden | France | Spain |
| --- | --- | --- | --- | --- | --- |
| Estonia | **0.17** | 0.45 | 0.52 | 1.42 | 2.09 |
| Åland | 0.23 | **0.46** | 0.48 | 1.69 | 2.36 |
| Sweden | 0.36 | 0.00 | **0.50** | 1.76 | 2.43 |
| France | 0.82 | 0.71 | 0.69 | **0.67** | 3.33 |
| Spain | 0.93 | 0.82 | 0.83 | 0.90 | **0.00** |
